# Short-Term Beetroot Juice Supplementation Enhances Strength, Reduces Fatigue, and Promotes Recovery in Physically Active Individuals: A Randomized, Double-Blind, Crossover Trial

**DOI:** 10.3390/nu17101720

**Published:** 2025-05-19

**Authors:** Atef Salem, Achraf Ammar, Mohamed Kerkeni, Mohamed Ali Boujelbane, Ayse Merve Uyar, Leonard Moritz Köbel, Saranya Selvaraj, Reza Zare, Katie M. Heinrich, Haitham Jahrami, Slim Tounsi, Piotr Zmijewski, Wolfgang I. Schöllhorn, Khaled Trabelsi, Hamdi Chtourou

**Affiliations:** 1 High Institute of Sport and Physical Education of Sfax, University of Sfax, Sfax 3000, Tunisia; asalem@uni-mainz.de (A.S.); mboujelb@uni-mainz.de (M.A.B.); trabelsikhaled@gmail.com (K.T.); h_chtourou@yahoo.fr (H.C.); 2 Department of Training and Movement Science, Institute of Sport Science, Johannes Gutenberg-University Mainz, 55122 Mainz, Germany; auyar@students.uni-mainz.de (A.M.U.); lkoebel@students.uni-mainz.de (L.M.K.); wolfgang.schoellhorn@uni-mainz.de (W.I.S.); 3 Research Laboratory, Molecular Bases of Human Pathology, LR19ES13, Faculty of Medicine of Sfax, University of Sfax, Sfax 3000, Tunisia; 4 Research Laboratory, Education, Motricity, Sport and Health, EM2S, LR19JS01, High Institute of Sport and Physical Education of Sfax, University of Sfax, Sfax 3000, Tunisia; 5 Department of Chemistry, Faculty of Applied Sciences, University of Sri Jayewardenepura, Gangodawila, Nugegoda, Colombo 10250, Sri Lanka; saranyaselvaraj.tmp@sjp.ac.lk; 6 SRH Campus Hamburg, SRH University of Applied Sciences Heidelberg, 20095 Hamburg, Germany; reza.zare.official73@gmail.com; 7 Department of Kinesiology, Kansas State University, Manhattan, KS 66506, USA; kmhphd@ksu.edu; 8 Department of Research and Evaluation, The Phoenix, Denver, CO 80205, USA; 9 Government Hospitals, Manama P.O. Box 12, Bahrain; 10 Department of Psychiatry, College of Medicine and Medical Sciences, Arabian Gulf University, Manama P.O. Box 26671, Bahrain; 11 Laboratory of Biopesticides (LBPES), Center of Biotechnology of Sfax, University of Sfax, Sfax 3000, Tunisia; slim.tounsi@cbs.rnrt.tn; 12 Department of Biomedical Sciences, Jozef Pilsudski University of Physical Education in Warsaw, 00-809 Warsaw, Poland; piotr.zmijewski@insp.pl; 13 Department of Movement Sciences and Sports Training, School of Sport Science, The University of Jordan, Amman 11942, Jordan; 14 Research Unit, Physical Activity, Sport, and Health, UR18JS01, National Observatory of Sport, Tunis 1003, Tunisia

**Keywords:** nitrate, resistance training, muscle oxygenation, heart rate variability, delayed onset muscle soreness

## Abstract

**Background/Objectives:** Beetroot juice (BJ), a natural source of dietary nitrate, has gained increasing attention for its potential to improve exercise performance and cardiovascular function. While its benefits are well documented in endurance contexts, less is known about its short-term effects on resistance training performance and recovery. Thus, this study investigated the effects of short-term BJ supplementation on strength performance, cardiovascular responses, muscle oxygenation, and post-exercise recovery in resistance-trained males. **Methods:** Twelve healthy men (age: 21.3 ± 1.9 years; body mass index: 21.42 ± 2.36 kg/m^2^) completed two supplementation protocols involving BJ, providing 450 mg of nitrate per day, and a nitrate-free placebo (PLA). Each protocol consisted of two laboratory visits, one to assess the acute ergogenic effects and another to evaluate recovery after 72 h, resulting in a total of four sessions over a two-week period. During the three consecutive days of supplementation, participants ingested a single 900 mL dose (15 g BJ powder/PLA) 2 h before the first session, followed by three daily 300 mL doses (5 g BJ each/PLA) over the next two days, and a final dose (15 g BJ powder/PLA) taken 2 h before the second session (72 h post-first session). Each testing session involved incremental back squat (BS) and bench press (BP) exercises at 60%, 70%, and 80% of the one-repetition maximum (1RM) performed to failure, with three-minute rest intervals between sets. Repetition to failure, movement velocity, peak power, peak heart rate (HR), and muscle oxygenation (SmO_2_) were recorded during BP and BS exercises. Heart rate variability (HRV) and blood lactate were assessed before and after each training session. Lower-limb strength (CMJ and SJ) and delayed-onset muscle soreness (DOMS) were assessed daily during the 3-day supplementation period. **Results:** BJ significantly increased repetitions completed at 80% 1RM during BP and BS (*p* < 0.05) compared to the PLA. Peak movement velocity improved across all intensities using BJ with higher values compared to the PLA at 60–80% 1RM (*p* < 0.05). SmO_2_ was higher in BJ at 70–80% 1RM) and further improved after 72 h of BJ supplementation (*p* < 0.05). Cardiovascular strain was reduced in BJ, evidenced by lower peak HRs and smaller post-exercise declines in HRV indices (*p* < 0.05). Post-exercise recovery favored BJ, with faster recovery in jump performance at 24 h and reduced upper-limb DOMS at 24–48 h (*p* < 0.05). **Conclusions:** Short-term BJ supplementation enhances high-intensity resistance performance, improves muscle oxygenation, attenuates cardiovascular strain, and accelerates neuromuscular recovery. These benefits highlight its potential as a practical strategy for athletes seeking to optimize training performance and recovery during periods of intense resistance training.

## 1. Introduction

Beetroot juice (BJ), rich in dietary nitrate (NO_3_^−^), has garnered increasing interest for its ergogenic potential in both sports performance and general cardiovascular health [1]. In sports nutrition, BJ supplementation is primarily attributed to its high NO_3_^−^ content, which, upon ingestion, follows a complex pathway. Upon ingestion, NO_3_^−^ is reduced to nitrite (NO_2_^−^) by oral bacteria and further converted into nitric oxide (NO) under hypoxic conditions, such as those during intense exercise [2,3,4]. Under hypoxic conditions such as those encountered during high-intensity resistance exercise, NO_2_^−^ is further reduced to nitric oxide (NO), a potent vasodilator that improves muscle blood flow, optimizes oxygen delivery, and enhances mitochondrial efficiency by reducing the oxygen cost of adenosine triphosphate (ATP) production [5,6].

Although BJ has been extensively studied in endurance sports (e.g., reductions in oxygen consumption), its application in resistance training (RT) remains less explored. Emerging evidence suggests that acute BJ supplementation (6.4–13 mmol NO_3_^−^) improves muscular endurance, power output, and repetition volume in exercises like the bench press and back squat, with pronounced effects in lower-body movements due to greater muscle mass engagement [6,7,8,9]. Multi-day supplementation (≥6 days) may amplify the benefits by elevating plasma NO_2_^−^, enhancing calcium sensitivity, and sustaining performance across sets [6,7]. These effects align with velocity-based training paradigms, where BJ’s ability to preserve movement velocity could optimize sport-specific adaptations [10].

Moreover, BJ has shown promising effects in modulating cardiovascular responses during exercise. Its vasodilatory properties not only facilitate increased blood flow and improved lactate clearance but may also contribute to blood pressure regulation and enhanced endothelial function [9,10]. BJ’s cardiovascular effects extend beyond performance, including enhanced lactate clearance, blood pressure regulation, and endothelial function [11,12]. Its vasodilatory properties also modulate heart rate variability (HRV), reflecting improved autonomic balance and recovery—key metrics for athlete monitoring [13,14].

Furthermore, BJ-derived NO is a key modulator of heart rate variability (HRV), an established marker of autonomic nervous system function and recovery [15]. HRV reflects the dynamic interplay between sympathetic and parasympathetic activity and serves as a valuable tool for monitoring internal load and recovery status in athletes [16]. Advanced HRV analysis—using methods such as the root mean square of successive differences (RMSSD)—has been validated in various sports settings, providing insights into parasympathetic activation and neuromuscular recovery post-exercise [17]. BJ has also been recognized for its broader health benefits, including cardiovascular diseases, through improvements in vascular endothelial function and blood pressure regulation [18]. In the context of RT, these cardiovascular adaptations may support enhanced lower-limb strength and reduced muscle soreness by optimizing muscle perfusion and accelerating recovery processes [19].

However, several gaps remain in the current literature. Most studies have either focused on the acute or prolonged effects of BJ supplementation, with limited research exploring the combined effects of acute ingestion followed by short-term use (e.g., 3 days). This supplementation approach may be particularly relevant for athletes who perform multiple high-intensity sessions per week and seek immediate performance and recovery benefits. The practical relevance is especially pronounced for strength athletes, who often prioritize rapid power output and neuromuscular recovery between sessions, compared to endurance athletes, who may benefit more from sustained aerobic efficiency. Furthermore, while the majority of BJ research has focused on endurance modalities, emerging evidence underscores its potential to enhance RT performance through improved muscular strength, power, and recovery. Nevertheless, while available strength-related studies emphasize performance outcomes, they often neglect underlying physiological responses like HRV and muscle oxygenation—critical for monitoring internal load and recovery. For strength athletes, understanding these mechanisms could optimize training periodization, whereas endurance athletes may prioritize BJ’s oxygen utilization benefits, underscoring the need for modality-specific research frameworks.

Thus, there is a need to clarify whether short-term BJ supplementation can provide both performance and recovery benefits while modulating cardiovascular and neuromuscular responses. Considering these observations, this study aimed to examine the effect of short-term BJ supplementation on strength performance, HRV responses, and muscle oxygenation, while exploring its recovery effects on lower-limb strength and muscle soreness. The hypotheses were as follows: (i) short-term BJ supplementation would enhance strength performance, (ii) short-term BJ supplementation would positively influence heart rate variability (HRV) responses, and (iii) short-term BJ supplementation would improve recovery by enhancing lower-limb strength, reducing muscle soreness, and improving muscle oxygenation.

## 2. Materials and Methods

### 2.1. Population

The minimum required sample size was calculated using the software G*power (version 3.1.9.6; Kiel University, Kiel, Germany) using the F test family (repeated measures, within factors). We set the values of α and power (1 − β) at 0.05 and 0.95, respectively. The effect size, based on Ranchal-Sanchez et al. [11] and discussed between authors was estimated to be 0.5 (medium effect). The minimum sample size required was nine participants for this study.

Eleven physically active males (age: 21.3 ± 1.9 years; BMI: 21.42 ± 2.36 kg/m^2^; 1RM BS: 97 ± 14.18 kg; 1RM BP: 36 ± 4.59 kg) were recruited. All had prior RT experience (≥3 sessions/week for ≥6 weeks) and were familiar with BP and BS exercises. Exclusion criteria included medication use affecting muscle biology, prior creatine supplementation, kidney or liver conditions, low blood pressure, or musculoskeletal injuries. Compliance with these criteria was confirmed via interviews. Participants had to follow dietary guidelines, avoid strenuous activity, and refrain from nonsteroidal anti-inflammatory drugs (NSAIDs). Informed consent was obtained, and ethical approval was granted by the local Research Ethics Committee of High Institute of Sport and Physical Education of El Kef, El Kef, Tunisia (ISSEPK-0033/2024). The study was registered at the Pan African Clinical Trials Registry database on 15 May 2025 (PACTR202505827886996).

### 2.2. Experimentation Protocol

**Figure 1:**
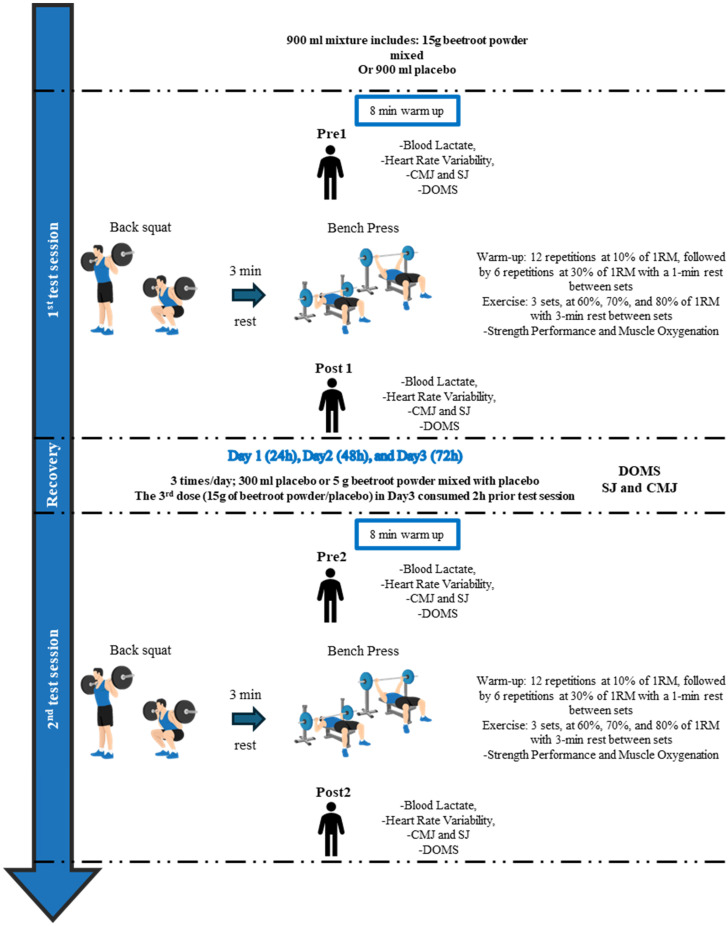
Experimental design. 1RM: one-repetition maximum; Pre1: before the 1st session; Post1: after the 1st session; Pre2: before the 2nd session; Post2: after the 2nd session; CMJ: countermovement jump; SJ: squat jump; DOMS: delayed onset of muscle soreness.

The study followed a double-blind, randomized, crossover, within-subject controlled design. During the first visit, participants underwent body composition assessment via bioimpedance (Tanita MC-780MA, Tokyo, Japan) and completed a familiarization protocol to standardize lifting techniques for BS and BP. To determine their one-repetition maximum (1RM), participants followed a warm-up protocol based on prior research investigating BJ effects on strength [7,11,14], adhering to the American Society of Exercise Physiologists (ASEP) guidelines for muscular strength assessment [20]. Both exercises were tested on the same day using a structured protocol: five repetitions at a 50% estimated 1RM, three repetitions at 70%, and up to five incremental 1RM attempts, each separated by 3 min rest intervals. Movement velocity was monitored [21,22] to confirm that 1RM loads aligned with expected velocities for maximal effort. These values were used to calibrate intensities for subsequent testing. Following the first visit, which included familiarization and 1RM determination, the protocol involved four testing sessions conducted over a two-week period, with at least 72 h of rest between sessions to ensure adequate recovery [23]. Testing occurred in the afternoon [24,25] under controlled environmental conditions (24 °C ± 1 °C) at a consistent time (±0.5 h) to minimize circadian effects [26,27]. Participants completed two supplementation protocols (BJ and placebo (PLA)), each involving two laboratory visits: one to assess acute ergogenic effects and another to evaluate 72 h recovery (Figure 1). Performance metrics—including incremental strength test outcomes (repetitions at 60%, 70%, 80% 1RM), peak HR, and muscle oxygen saturation (SmO_2_)—were recorded during BP and BS exercises. HRV indices and blood lactate were measured before and after the 1st and 2nd testing sessions. Recovery was tracked via countermovement jump (CMJ), squat jump (SJ), and upper (elbow flexor) and lower-limb (knee extensor) delayed onset muscle soreness (DOMS) assessed before and after the 1st testing session, at 24 h, at 48 h, and before and after the 2nd testing session (Figure 1).

### 2.3. Supplementation Protocol

Participants consumed Bio beetroot powder (Beta vulgaris; GymBeam GmbH, Berlin, Germany) dissolved in a nitrate-free placebo juice that matched in color, texture, and nutritional content, except for the absence of nitrate. The beetroot powder’s nitrate content and purity (7.26 mmol or 450 mg NO_3_^−^ per 15 g) were confirmed by independent lab testing at CBS, University of Sfax, Tunisia. Detailed nutritional facts of the used beetroot powder are provided in Table 1. The placebo (PLA) was nitrate-free but nutritionally matched to beetroot juice (identical calories, carbs, sugars; Table 1). Two individuals, who were not involved in other study procedures, were responsible for randomizing participants and preparing supplement kits. These kits contained the assigned supplement, detailed usage instructions, measuring spoons, and a water bottle. The specific order in which participants received either BJ or the PLA was only revealed to researchers after all data had been collected. On the first day, participants took the full 15 g dose (in 900 mL of placebo juice) two hours before testing. Over the next two days, they consumed three 5 g servings (each in 300 mL juice) spaced throughout the day, with the final dose taken two hours before the second testing session (72 h later). This protocol ensured a consistent nitrate intake (i.e., 450 mg NO_3_^−^ per day) sufficient to produce ergogenic effects [2,28].

Participants were required to abstain from food or beverages (except for water, which was allowed as needed) for at least two hours before each test to ensure accurate assessments. To maintain dietary consistency, participants completed a 24 h dietary recall before each test session [14]. These recalls were verbally conducted and recorded by a trained researcher to identify any deviations from dietary instructions, with particular attention to nitrate intake and the consumption of substances that could interfere with oral nitrate reduction. Further guidelines for participants included the following: (i) avoiding nitrate-rich foods (e.g., beetroot, celery, spinach) for 48 h before each testing session to control background dietary nitrate; (ii) avoiding foods high in stimulants (e.g., caffeine), gum, sweets, and alcohol, which could influence the oral microbiota for three days before testing; (iii) refraining from brushing their teeth on the morning of the test and avoiding antiseptic mouth rinses for at least one week before and throughout the study, as these could inhibit the conversion of nitrate to nitrite [29]; (iv) ensuring proper hydration; (v) avoiding intense physical activity; and (vi) sleeping at least eight hours each night. Compliance with these guidelines was emphasized during the initial briefing sessions, and adherence was verbally confirmed before each testing session.

### 2.4. Strength Exercise Protocol

During each testing session, participants started with an 8 min standardized warm-up under supervision, which included light muscle activation on a treadmill. After warming up, they performed the BS first, followed by the BP, both executed using a Smith machine, with a 3 min rest between exercises. Before the incremental strength test, participants completed a structured warm-up for both exercises, consisting of 12 repetitions at 10% of their 1RM, followed by 6 repetitions at 30% of their 1RM, with a 1 min rest between sets [11]. Afterward, they rested for 2 min before the test began. The incremental strength test comprised three sets with progressively increasing loads at 60%, 70%, and 80% of their 1RM, with repetitions performed until muscle failure. A 3 min rest was provided between sets to allow sufficient recovery and maintain performance [30]. Both BS and BP were performed through a full range of motion [31]. Participants were instructed to execute the concentric phase of each repetition at maximum velocity to enhance muscle strength development [22]. The full testing session, including the warm-up, lasted about 35 min.

### 2.5. Measurements

#### 2.5.1. Incremental Strength Test and Performance

Maximum repetitions completed at 60%, 70%, and 80% RM until failure were noted for both the BP and BS. The maximum velocity (MV) and maximum power (MP) per set were recorded using the validated accelerometer-based sensor “Vmax Pro” (Blaumann & Meyer, Sports Technology UG, Magdeburg, Germany), which demonstrated high agreement with Vicon (R^2^ = 0.935) and T-Force (R^2^ = 0.968) systems during squat movements [32]. Execution velocity and power output were continuously monitored by the researchers.

#### 2.5.2. Heart Rate Variability (HRV) Analysis

HRV was assessed at 5 min pre-session and 5 min post-session. Data were collected via a Polar H10 heart rate monitor and analyzed using the Elite HRV app (version 5.5.8) [33]. Time-domain parameters included the mean RR interval (MeanRR), root mean square of successive differences (RMSSD), and standard deviation of RR intervals (SDNN). Frequency-domain analysis evaluated low-frequency (LF) and high-frequency (HF) components. Peak heart rate during exercise was also recorded.

#### 2.5.3. Muscle Oxygenation

Muscle oxygenation was assessed using Near-Infrared Spectroscopy (NIRS), a non-invasive method to monitor oxygen delivery and consumption in muscle tissue. This technique provided semi-quantitative data on oxygen levels in hemoglobin/myoglobin (tissue O_2_ stores) and total hemoglobin (THb) concentration, reflecting blood volume changes [34]. Real-time SmO_2_ was measured using the validated Moxy 3-Sensor Bundle (Fortiori Design LLC, Spicer, MN, USA) [35]. Additionally, THb was monitored to evaluate blood volume and flow dynamics in the muscle.

#### 2.5.4. Lower-Limb Strength Tests

CMJ and SJ performances were quantified via the My Jump 2 app [36]. For SJ, participants executed a maximal vertical jump from a 90° knee angle with hands on hips. For CMJ, jumps begin from a standing position without arm swing. Three attempts per test were allowed, with the highest-performing attempt retained for analysis.

#### 2.5.5. Blood Lactate Measurement

Lactate levels were assessed using Lactate Pro 2 (AKRAY Europe B.V., Amstelveen, The Netherlands). Samples were obtained from the sterilized earlobe (70% ethanol) following standardized protocols [37,38].

#### 2.5.6. Delayed-Onset Muscle Soreness (DOMS)

DOMS was evaluated using a visual analog scale (0–10) for knee extensors and elbow flexors. Scores were normalized to 100% of the maximum perceived soreness [25,39], where 0 indicated no soreness and 10 represented intolerable discomfort.

### 2.6. Statistical Analysis

All statistical procedures were performed using the R programming language (version 4.4.0) [40]. Descriptive statistics are presented as mean ± standard deviation (SD). The normality of the data was assessed and confirmed using the Shapiro–Wilk test. To examine the effects of supplementation condition, time, and intensity, a three-way repeated measures analysis of variance (ANOVA) with Greenhouse–Geisser correction was conducted. When significant main or interaction effects were detected, post hoc pairwise comparisons with Bonferroni adjustments were performed. Delta change (∆pre-post) was calculated as follows: ∆pre-post (%) = ((score at post-session − score at pre-session)/score at pre-session) × 100. To assess differences in HRV indices between supplementation conditions and time effects, a two-way ANOVA was conducted, followed by post hoc pairwise comparisons with Bonferroni adjustments. Moreover, to assess recovery effects in CMJ, SJ, and DOMS between supplementation conditions and time effects, a two-way ANOVA was conducted, followed by post hoc pairwise comparisons with Bonferroni adjustments. Effect sizes were calculated as partial eta-squared (η^2^p), with small (0.01), moderate (0.06), and large (0.14) thresholds [41]. Standardized effect size (Cohen’s d) was used to interpret the magnitude of mean differences and classified according to Hopkins [42]: trivial (d ≤ 0.20), small (0.20 < d ≤ 0.60), moderate (0.60 < d ≤ 1.20), large (1.20 < d ≤ 2.0), very large (2.0 < d ≤ 4.0), and extremely large (d > 4.0). A significant level of *p* < 0.05 was used for all analyses. Normality was assessed using the Shapiro–Wilk test with the “rstatix” package (version 0.7.2) [43], and ANOVA for normally distributed data was conducted with the “afex” package (version 1.3-1) [44]. Pairwise comparisons were performed using the “emmeans” package (version 1.10.2) [45], and visualizations were generated with the “ggplot2” package (version 3.5.2) [46].

## 3. Results

### 3.1. Dietary Intake

Dietary intake for both BJ and PLA conditions are presented in Table 2. No significant differences in energy or macronutrient intake were found between conditions (*p* > 0.05).

### 3.2. Strenght Performance and Physiological Parameters

All three-way ANOVA results for reached repetitions, peak velocity, peak power, peak HR, SmO_2_, and tHb are presented in Table S1.

#### 3.2.1. Reached Repetitions

**Figure 2:**
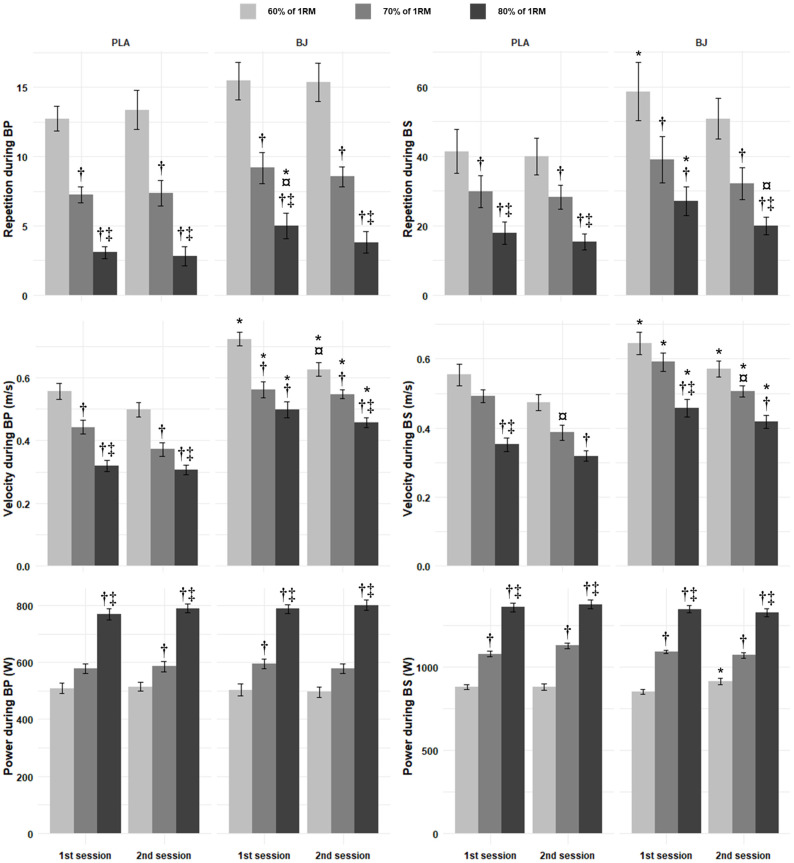
Reached repetition, peak velocity, and peak power recorded during bench press (BP) and back squat (BS) incremental tests in two sessions under beetroot juice (BJ) and placebo (PLA) conditions. †: Significantly different compared to 60% RM; ‡: significantly different compared to 70% RM; *: significantly different compared to PLA; ¤: significantly different compared to 1st session.

Regarding the reached repetitions during BP, there were significant main effects of condition and intensity (Table S1). In all conditions and sessions, the pairwise comparisons showed that performance values increased significantly from 60% to 70% RM, 60% to 80% RM, and 70% to 80% RM (*p* < 0.001, d = 1.63 to 3.97; Figure 2). The reached repetitions for 80% RM in BJ were significantly lower in the second than the first session (*p* = 0.046, d = 0.69; Figure 2). Moreover, BJ presented higher values than the PLA, with significant differences observed at 80% RM (*p* = 0.0344, d = 0.74) in the first session, as well as at 70% RM in second session (*p* = 0.0189, d = 0.84) (Figure 2). During the BS, there were significant main effects of condition, session, and intensity (Table S1). The reached repetitions in BS increased significantly from 60% to 70% RM, 60% to 80% RM, and 70% to 80% RM (*p* < 0.001, d = 0.98–2.55; Figure 2) under all conditions and in all sessions, except from 70% to 80% RM in the first session for BJ (*p* = 0.1489, d = 0.63). BJ repetitions in the second session were significantly lower compared to those in the first session (*p* = 0.0126, d = 0.91) for 80% RM (Figure 2). BJ was reported to have significant higher repetitions compared to the PLA at 60% and 80% RM in the first session (*p* = 0.0104 and 0.0069, d = 0.95 and 096, respectively) (Figure 2).

#### 3.2.2. Peak Velocity

Concerning velocity during BP, significant main effects of condition, session, and intensity were observed (Table S1). In all conditions and all sessions, velocity decreased significantly from 60% to 70% RM (*p* < 0.05, d = 0.86–1.72), 60% to 80% RM (*p* < 0.002, d = 1.78–2.21), and 70% to 80% RM (*p* < 0.05, d = 1.06–1.57), except from 70% to 80% RM in the first session for BJ (*p* = 0.395, d = 0.48) (Figure 2). In those given BJ, velocity at 60% RM was significantly lower in the second than the first session (*p* = 0.0114, d = 0.93; Figure 2). BJ presented significantly greater velocities at all intensity levels and sessions (*p* < 0.011, d = 0.94–2.29; Figure 2). Regarding the velocity under BS supplementation, significant main effects of condition, session, and intensity were found (Table S1). The PLA’s velocity decreased significantly from 60% to 80% RM in both sessions (*p* = 0.0002 and 0.0003, and d = 2.03 and 1.88, respectively, for the first and second sessions), while velocity decreased only in the first session from 70% to 80% RM (*p* = 0.0040, d = 1.33) (Figure 2). Similarly, velocity in the BJ decreased significantly from 60% to 80% RM across the first (*p* = 0.0026, d = 1.41) and the second sessions (*p* = 0.0029, d = 1.39), while a significant decrease was found from 70% to 80% RM in the first session (*p* = 0.0001, d = 2.13) (Figure 2). Moreover, velocity was significantly lower in the first compared to the second session in 70% RM for both the PLA (*p* = 0.0062, d = 1.04) and BJ (*p* = 0.0211, d = 0.82) (Figure 2). Additionally, BJ supplementation resulted in significantly higher velocity than the PLA did across all intensity levels in both the first and the second sessions (*p* < 0.05, d = 0.69–1.3) (Figure 2).

#### 3.2.3. Peak Power

Concerning the peak power during BP supplementation, a significant main effect of intensity was found (Table S1). In the PLA condition, the power significantly increased from 60% to 80% RM, and from 70% to 80% RM (*p* < 0.0001, d = 2.35–4.77) in both sessions, while it increased from 60% to 70% RM in the second session only (*p* = 0.0073, d = 1.21) (Figure 2). Similarly for BJ, there were significant increases in power from 60% to 80% RM and from 70% to 80% RM (*p* < 0.0001, d = 2.35–3.94) across both sessions (Figure 2). However, BJ showed a significant increase from 60% to 70% RM in the first session only (*p* = 0.0442, d = 0.89) (Figure 2). Regarding the peak power during BS, there was a significant main effect of intensity and a significant condition × session × intensity interaction (Table S1). In all conditions and all sessions, power increased significantly from 60% to 70% RM (*p* < 0.0005, d = 1.8–4.19), 60% to 80% RM (*p* < 0.0001, d = 4.24–6.03), and 70% to 80% RM (*p* < 0.0001, d = 2.25–3.94) (Figure 2). Additionally, peak power significantly increased from the first to second session for performance under BJ supplementation at 60% RM (*p* = 0.0083, d = 0.99) and for performance on the PLA at 70% RM (*p* = 0.0367, d = 0.73) (Figure 2).

#### 3.2.4. Peak HR

**Figure 3:**
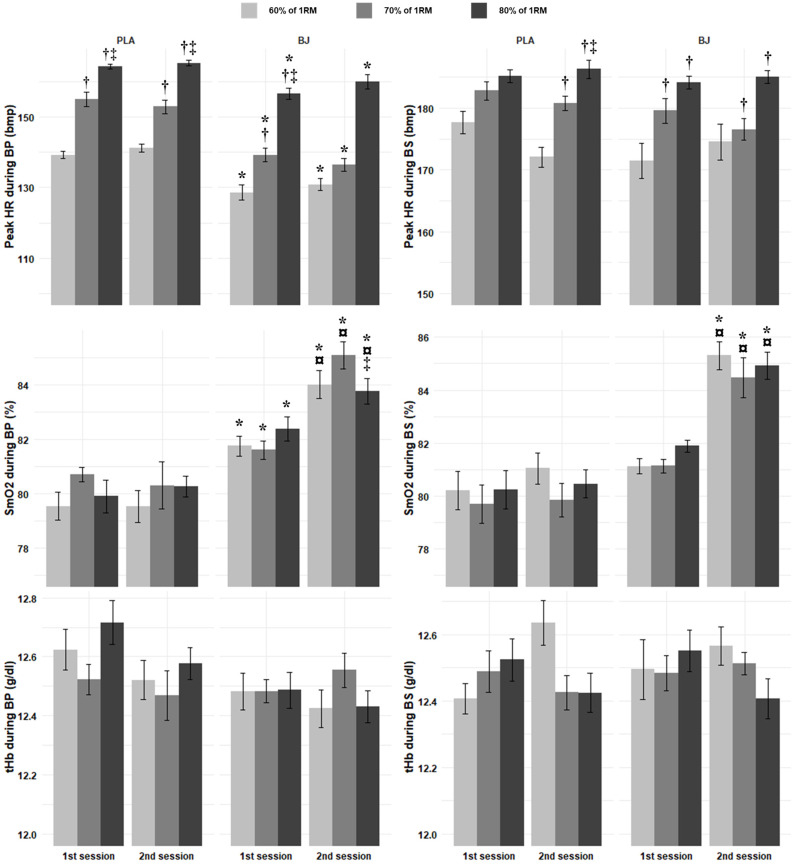
Peak heart rate (HR), muscle oxygenation (SmO2), and total hemoglobin (tHb) recorded during bench press (BP) and back squat (BS) incremental tests at two sessions for beetroot juice (BJ) and placebo (PLA) conditions. †: Significantly different compared to 60% RM; ‡: significantly different compared to 70% RM; *: significantly different compared to PLA; ¤: significantly different compared to 1st session.

Regarding the peak HR recorded during the BP, there were significant main effects of condition and intensity (Table S1). Additionally, a significant condition × intensity interaction was found (Table S1). In both conditions, HR increased with increasing intensity (*p* < 0.0001, d = 0.78–6.84), except from 60% to 70% RM for BJ (*p* = 0.1304, d = 0.78) during the second session (Figure 3). Additionally, the PLA produced higher HRs compared to BJ across all intensity levels in both sessions (*p* < 0.05, d = 1.18–2.08) (Figure 3).

During the BS, there were significant main effects of condition and intensity; also, a significant condition × session × intensity interaction was detected (Table S1). For the PLA, during the second session, HRs significantly increased from 60% to 70% RM (*p* = 0.0014, d = 1.99), 60% to 80% RM (*p* = 0.0003, d = 3.02), and 70% to 80% RM (*p* = 0.0370, d = 1.14) (Figure 3). Similarly, during the first session, BJ was reported to lead to significant increases from 60% to 70% RM (*p* = 0.0314, d = 1.09) and from 60% to 80% RM (*p* = 0.0066, d = 1.16) (Figure 3). In contrast, for BJ, during the 2nd session, HRs significantly increased from 60% to 80% RM (*p* = 0.0307, d = 0.99) and 70% to 80% RM (*p* = 0.0137, d = 1.13) (Figure 3).

#### 3.2.5. SmO_2_

During BPs, there were significant main effects of condition and session, as well as a significant condition × session interaction (Table S1). BJ supplementation during the second session showed a significant decrease in SmO_2_ from 70% to 80% RM (*p* = 0.478, d = 0.87) (Figure 3). Additionally, BJ’s SmO_2_ was significantly higher in the second session compared to the first session for 70% (*p* = 0.0001, d = 2.03) and 80% RM (*p* = 0.0187, d = 0.85) (Figure 3). BJ led to higher SmO_2_ compared to the PLA during the first and second sessions, at 60% (*p* = 0.0048 and 0.0001, d = 1.09 and 1.94, respectively), 70% (*p* = 0.0169 and 0.0003, d = 0.86 and 1.61, respectively), and 80% RM (*p* = 0.0004 and 0.0001, d = 1.55 and 1.8, respectively) (Figure 3).

The analysis for SmO_2_ during BSs revealed significant main effects of the condition and session, as well as a significant condition × session interaction (Table S1). SmO_2_ under BJ supplementation showed a significant increase from the first to the second session at 60% (*p* < 0.0001, d = 10.24), 70% (*p* = 0.0009, d = 4.68), and 80% RM (*p* = 0.0004, d = 5.28) (Figure 3). Additionally, SmO_2_ was significantly higher under BJ supplementation during the second session compared to the PLA at 60% (*p* < 0.0001, d = 11.27), 70% (*p* = 0.0001, d = 6.57), and 80% RM (*p* = 0.0001, d = 5.94) (Figure 3).

#### 3.2.6. Total Hemoglobin

Regarding the tHb during BPs, a significant main effect of condition was observed (Table S1), where tHb was significantly lower for those taking BJ compared to those on the PLA during ST at 80% RM (*p* = 0.0232, d = 0.81). Concerning tHb during BSs, there was a significant session × intensity interaction (Table S1), where a significant increase in tHb at 80% RM from AC to ST was revealed (*p* = 0.0266, d = 0.78) (Figure 3).

### 3.3. ∆Pre-Post Change in HRV Indices and Lactate

The ∆Pre-Post change in HRV indices and the lactate and ANOVA results are presented in Table 3. For MeanRR, there was a significant main effect of session, where the PLA and BJ showed a significant decrease from AC to ST (*p* = 0.0113 and 0.0123, d = 0.93 and 0.92, respectively). Similarly, RMSSD showed a significant main effect of session, with the PLA and BJ showing a significant decrease from AC to ST (*p* = 0.0027 and 0.021, d = 1.2 and 0.832, respectively), with a greater ∆Pre-Post change for the PLA compared to that for BJ at AC (*p* = 0.044, d = 0.69). For SDNN, no significant effects were found. Additionally, LF and HF showed a significant main effect of session, with significant decreases in HF changes for the PLA and BJ from AC to ST (*p* = 0.0025 and 0.0045, d = 1.21 and 1.1, respectively). Lastly, for lactate, there were no significant effects.

### 3.4. Post-Session Recovery

**Figure 4:**
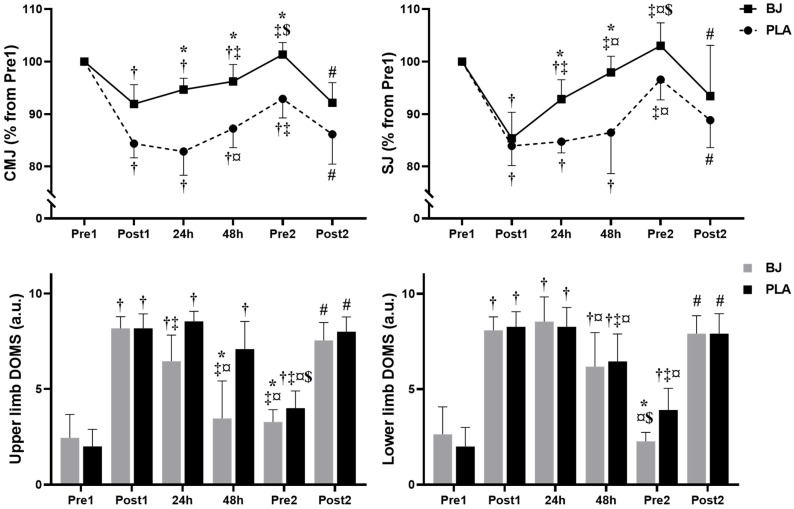
Countermovement jump (CMJ), squat jump (SJ), and delayed onset of muscle soreness (DOMS) recorded before and after the 1st session, at 24 h, at 48 h, and before and after the 2nd session for the beetroot juice (BJ) and placebo (PLA) conditions. †: significantly different compared to Pre1; ‡: significantly different compared to Post1; ¤: significantly different compared to 24 h; $: significantly different compared to 48 h; #: significantly different compared to Pre2; *: significantly different compared to PLA.

CMJ, SJ, and upper- and lower-limb DOMS results are visualized in Figure 4.

#### 3.4.1. CMJ

The analysis showed no significant effects of condition (F(1, 10) = 4.42, *p* = 0.062, η^2^p = 0.306). However, there was a significant main effect of time (F(3.13, 31.26) = 91.38, *p* < 0.001, η^2^p = 0.901) and a condition × time interaction (F(2.21, 22.06) = 17.61, *p* < 0.001, η^2^p = 0.638). In the PLA, CMJ performance significantly decreased from Pre1 to Post1, (*p* < 0.0001, d = 5.55), and CMJ performance at Pre1 was significantly higher compared to that at 24 h, 48 h, and Pre2 (*p* < 0.0008, d = 2.03–4.07). However, CMJ performance significantly increased from Post1 to Pre2 (*p* = 0.0006, d = 2.5) and from 24 h to 48 h (*p* = 0.0021, d = 1.79). Also, CMJ performance significantly decreased from Pre2 to Post2 (*p* = 0.0008, d = 2.03). With BJ supplementation, CMJ performance decreased significantly from Pre1 to Post1 (*p* = 0.0002, d = 2.45), 24 h (*p* = 0.0001, d = 2.47), and 48 h (*p* = 0.0220, d = 1.31). CMJ performance also increased significantly from Post1 to 48 h (*p* < 0.0001, d = 3.09) and Pre2 (*p* < 0.0001, d = 4.74), and from 48 h to Pre2 (*p* < 0.0001, d = 3.5). There was also a significant decrease for CMJ performance under BJ supplementation from Pre2 to Post2 (*p* < 0.0001, d = 2.99). Otherwise, BJ led to higher CMJ performance compared to the PLA at 24 h (*p* = 0.0123, d = 0.92), 48 h (*p* = 0.0364, d = 0.73), and Pre2 (*p* = 0.0442, d = 0.69).

#### 3.4.2. SJ

There was no significant effect of condition (F(1, 10) = 3.28, *p* = 0.1, η^2^p = 0.247). However, there was a significant main effect of time (F(2.66, 26.61) = 42.39, *p* < 0.001, η^2^p = 0.809) and a condition × time interaction (F(2.64, 26.42) = 6.40, *p* = 0.003, η^2^p = 0.390). In the PLA condition, SJ performance significantly decreased from Pre1 to Post1 (*p* < 0.0001, d = 3.63), 24 h (*p* < 0.0001, d = 5.99), and 48 h (*p* = 0.0085, d = 1.5). Additionally, performance was significantly increased from Post1 to Pre2 (*p* = 0.0001, d = 2.7) and from 24 h to Pre2 (*p* < 0.0001, d = 3.06). SJ performance decreased significantly from Pre2 to Post2 (*p* = 0.0078, d = 1.15). In BJ conditions, SJ performance significantly decreased from Pre1 to Post1 (*p* < 0.0001, d = 4.51) and at 24 h (*p* = 0.0007, d = 2.06). Furthermore, performance was significantly lower at Post1 compared to 24 h (*p* = 0.0004, d = 2.18), 48 h (*p* < 0.0001, d = 4.59), and Pre2 (*p* < 0.0001, d = 4.72), at 24 h compared to that at 48 h and Pre2 (*p* < 0.0001, d = 2.96 and 3.23), as well as at 48 h compared to that at Pre2 (*p* = 0.0020, d = 1.81). Furthermore, SJ performance under BJ conditions significantly decreased from Pre2 to Post2 (*p* = 0.0349, d = 1.22). Moreover, BJ led to higher SJ performance compared to the PLA at 24 h (*p* = 0.0449, d = 0.69) and 48 h (*p* = 0.0105, d = 0.94).

#### 3.4.3. Upper-Limb DOMS

There were significant main effects of condition (F(1, 10) = 34.53, *p* < 0.001, η^2^p = 0.775) and time (F(2.63, 26.28) = 139.78, *p* < 0.001, η^2^p = 0.933), as well as a significant condition × time interaction (F(1.74, 17.40) = 9.59, *p* = 0.002, η^2^p = 0.490). DOMS for the PLA significantly increased from Pre1 to Post1 (*p* < 0.0001, d = 5.73), 24 h (*p* < 0.0001, d = 6.33), 48 h (*p* = 0.0001, d = 2.57), and Pre2 (*p* = 0.0034, d = 1.69). Additionally, DOMS was significantly higher at Post1 compared to Pre2 (*p* < 0.0001, d = 4), at 24 h compared to Pre2 (*p* < 0.0001, d = 4.4), and at 48 h compared to Pre2 (*p* = 0.0007, d = 2.07). DOMS significantly increased from Pre2 compared to Post2 (*p* < 0.0001, d = 4.45). Concerning the BJ condition, DOMS significantly increased from Pre1 to Post1 (*p* < 0.0001, d = 4.03), and 24 h (*p* = 0.0013, d = 1.92). Furthermore, DOMS was significantly higher at Post1 compared to 24 h (*p* = 0.0254, d = 1.26), 48 h (*p* = 0.0001, d = 2.89), and Pre2 (*p* < 0.0001, d = 5.22). DOMS under BJ supplementation at 24 h was also significantly higher compared to that at 48 h (*p* < 0.0001, d = 2.98) and Pre2 (*p* = 0.0028, d = 1.73). Furthermore, DOMS increased significantly from Pre2 to Post2 (*p* < 0.0001). Additionally, BJ led to lower DOMS compared to the PLA at 24 h (*p* = 0.0013, d = 4.39) and 48 h (*p* = 0.0016, d = 4.3).

#### 3.4.4. Lower-Limb DOMS

There was neither a significant main effect of condition (F(1, 10) = 1.10, *p* = 0.319, η^2^p = 0.099) nor a condition × time interaction (F(2.05, 20.55) = 2.53, *p* = 0.103, η^2^p = 0.202). However, there was a significant main effect of time (F(3.37, 33.71) = 153.04, *p* < 0.001, η^2^p = 0.939). In the PLA condition, DOMS significantly increased from Pre1 to Post1 (*p* < 0.0001, d = 5.68), 24 h (*p* < 0.0001, d = 6.94), 48 h (*p* < 0.0001, d = 3.44), and Pre2 (*p* = 0.0371, d = 1.21). However, DOMS significantly decreased from Post1 to 48 h (*p* = 0.0233, d = 1.29) and Pre2 (*p* < 0.0001, d = 3.39), and from 24 h to 48 h (*p* = 0.0035, d = 1.69) and Pre2 (*p* < 0.0001, d = 2.79). Additionally, DOMS increased from Pre2 to Post2 (*p* = 0.0001, d = 2.83). DOMS under BJ supplementation significantly increased from Pre1 to Post1 (*p* < 0.0001, d = 3.47), 24 h (*p* < 0.0001, d = 3.47), and 48 h (*p* = 0.0043, d = 1.64). Furthermore, DOMS decreased from 24 h to 48 h (*p* = 0.001, d = 1.96) and Pre2 (*p* < 0.0001, d = 4.41), and from 48 h to Pre2 (*p* = 0.0004, d = 2.22). Also, DOMS reported significant increases from Pre2 to Post2 (*p* < 0.0001, d = 6.96). Moreover, BJ led to lower DOMS compared to the PLA at Pre2 (*p* = 0.0036, d = 1.14).

## 4. Discussion

The primary aim of this study was to assess the impact of short-term BJ supplementation on strength performance, HRV responses, muscle oxygenation, and post-exercise recovery. Results demonstrated that BJ significantly enhanced strength performance, as indicated by higher repetitions and peak velocity at 80% 1RM during BPs and BSs, alongside attenuated cardiovascular strain, with lower peak HRs in the BJ condition compared to those for the PLA, and moderated autonomic stress with smaller reductions in HRV indices under BJ supplementation such as RMSSD and HF. Also, BJ led to a higher SmO_2_ after 3 days of ingestion compared to the PLA. While the BJ improved post-exercise recovery of lower-limb strength (CMJ and SJ) and reduced upper-limb DOMS, it did not mitigate lower-limb DOMS or lactate accumulation. These outcomes confirm the hypothesis that BJ enhances strength performance and partially supports its role in improving recovery and HRV regulation.

Reached repetitions during both exercises decreased at higher intensities following BJ supplementation, suggesting a potentially ergogenic effect likely driven by enhanced neuromuscular efficiency and delayed fatigue. This performance enhancement was especially evident during the second session, where BJ outperformed the PLA at higher loads—possibly due to improved phosphocreatine resynthesis and faster calcium kinetics in type II fibers, as supported by previous findings from trained female athletes and team-sport players [47,48]. Peak velocity also reduced with increasing intensity, with BJ consistently achieving greater velocities than the PLA. On the other hand, peak power increased with rising exercise intensity in both exercises, suggesting that the essential load–power relationship of the neuromuscular system may overshadow any small ergogenic contributions of nitrate supplementation [6,14]. Furthermore, BJ, rich in dietary nitrates, enhances muscle performance by increasing NO bioavailability, which improves vasodilation and muscle contraction kinetics, with its high nitrate content being converted into NO in the body, playing a crucial role in muscle function by increasing the maximal shortening velocity and peak power of muscle fibers [47,49,50]. This effect is thought to result from NO’s role in improving blood flow and reducing the oxygen cost of exercise, thereby enhancing exercise performance [51].

These improvements in strength performance coincided with cardiovascular adaptations; BJ significantly reduced peak HR compared to the PLA, indicating reduced cardiovascular strain. This HR response, particularly during BPs at 60–70% 1RM in the second session, may reflect an adaptive effect from repeated NO exposure, which enhances cardiovascular efficiency at submaximal loads [52]. For the BS, the attenuated HR increased with BJ supplementation—especially in the second session—suggesting that NO helped maintain muscle oxygenation under high mechanical stress, minimizing the need for cardiac compensation [48,53]. In contrast, the PLA showed a HR rise at higher intensities, emphasizing the cardiovascular system’s reliance on elevated cardiac output when peripheral perfusion is limited [54]. BJ appears to stabilize oxygen delivery, reducing acute HR-driven strain and potentially improving exercise tolerance [49,55]. Mechanistically, BJ’s ergogenic effects proceed from the NO_3_^−^–NO_2_^−^–NO pathway, where NO_3_^−^ is converted by oral bacteria and under hypoxia into NO, a potent vasodilator that enhances skeletal and coronary muscle perfusion, reduces systemic vascular resistance, and improves stroke volume [56,57]. Through the Frank–Starling mechanism, greater venous return stretches the myocardium, optimizing contractility and reducing the HR needed to maintain cardiac output. Simultaneously, NO enhances mitochondrial efficiency by reducing the oxygen cost of ATP production, thereby decreasing the metabolic drive for HR elevation [4]. These dual effects—vascular and mitochondrial—may explain the lower peak HR and improved performance with BJ. Beyond NO, BJ’s antioxidant and anti-inflammatory compounds (e.g., betalains and polyphenols) may mitigate oxidative stress and muscle damage, contributing to more efficient recovery and performance sustainability [58].

Furthermore, SmO_2_ responses supported the cardiovascular and performance findings, offering additional evidence of BJ’s vasoactive effects. During BPs, BJ elevated SmO_2_ across all intensities compared to the PLA, suggesting improved microvascular perfusion [4]. During the BS, SmO_2_ increases in BJ were observed only during the second session (after 3 days of supplementation), indicating a potential cumulative effect of repeated nitrate intake. This aligns with the nitrate–nitrite–NO pathway, in which dietary nitrate is reduced to nitrite by oral bacteria and further converted into NO in the bloodstream, particularly under hypoxic conditions typical of RT [3,59]. Repeated BJ ingestion resulted in greater NO availability, which in turn would have promoted vasodilation, improved capillary blood flow, and enhanced oxygen delivery to working muscles [4]. These vascular effects manifest as elevated SmO_2_, reflecting both improved oxygen supply and potentially more efficient oxygen extraction [60]. This is consistent with findings from endurance studies in which NO has been shown to optimize mitochondrial function and lower the oxygen cost of ATP production [61].

Importantly, HRV results demonstrated that BJ consumption reduced RMSSD from pre- to post-sessions, reflecting increased sympathetic dominance and internal workload. This transient decrease in RMSSD aligns with previous findings that RT typically suppresses parasympathetic modulation during intense exercise [62]. The higher total repetitions and HRs in the BJ further validate the existence of a state of increased physiological demand. Conversely, post-exercise, RMSSD returned to the baseline in both conditions, but with a high recovery slope in BJ, suggesting that BJ may enhance autonomic recovery through NO-induced improvements in vagal modulation, as supported by studies reporting accelerated parasympathetic recovery following strength exercise with BJ intake [61]. However, the absence of blood pressure changes and the short HRV measurement duration impose limitations on interpreting these findings fully, while the exercise order (BS preceding BP) might have introduced cumulative neuromuscular fatigue that altered HRV responses.

BJ supplementation significantly enhanced neuromuscular recovery, as evidenced by improved CMJ and SJ performance at 24 h and 48 h after the first session compared to the PLA. Following initial performance declines immediately post-exercise, BJ participants exhibited a more rapid return to near-baseline values after 72 h, demonstrating accelerated recovery kinetics [59,63]. These findings align with those in the literature, in which more than 60% of nitrate supplementation studies report improved recovery metrics, particularly in attenuating DOMS and restoring muscle function [64]. For example, upper-limb DOMS was notably reduced with BJ at 24 h and 48 h, likely due to NO-mediated anti-inflammatory effects and enhanced tissue perfusion—mechanisms supported by previous investigations showing nitrate-induced improvements in vascular function and inflammation modulation [65]. In contrast, lower-limb DOMS responses were less pronounced, which may reflect regional variability in NO bioavailability or differences in the muscle damage profile between upper and lower-limb exercise [66]. Furthermore, NO_3_^−^ from BJ may accelerate recovery by preserving muscle excitability, improving sarcoplasmic calcium release, and promoting mitochondrial biogenesis [67]; these pathways contribute to faster phosphocreatine resynthesis that is often not captured by traditional biomarkers such as creatine kinase [65]. Importantly, these effects seem to be dose-dependent, with optimal recovery benefits observed around moderate nitrate doses (approximately 8–12.9 mmol), while higher doses may yield diminishing returns [68]. Furthermore, the synergistic action of bioactive compounds such as betalains may confer additional antioxidant and anti-inflammatory support, thereby enhancing recovery outcomes compared to when nitrate is administered in the form isolated salts [66,69]. Recent studies have also noted that acute BJ ingestion does significantly reduce RPE, and it does lower markers associated with muscle damage, thereby enhancing neuromuscular efficiency during repeated high-intensity bouts [64]. These benefits, observed in conjunction with improvements in CMJ and SJ performance at 24 h and 48 h after the first session, suggest that BJ can facilitate more rapid autonomic and functional recovery likely mediated by enhanced NO-induced vasodilation and improved calcium handling [70]. However, biochemical and molecular biomarkers are warranted to fully elucidate the contribution of NO-mediated mechanisms versus the potential synergistic effects of other beetroot components [1,58,69]. Collectively, these acute outcomes complement prior evidence showing that chronic BJ supplementation (over 5–7 days) augments training adaptations by increasing resistance exercise repetitions and mitigating post-exercise muscle soreness, primarily through improved oxygen delivery and neuromuscular function [59].

While our findings support the ergogenic effects of short-term BJ supplementation (450 mg nitrate/day) on physical performance, physiological responses, and recovery, it is important to acknowledge emerging concerns regarding long-term or high-dose nitrate consumption. Elevated nitrate intake can increase the endogenous formation of N-nitroso compounds (NOCs), which are known to contribute to carcinogenic processes, particularly through DNA adduct formation such as O6-alkylguanine [71,72]. The nitrate dose used in our study was selected based on prior recommendations for optimizing ergogenic benefits in athletic populations [28], while still considering safety guidelines. Nitrates are widely considered an effective ergogenic aid when taken acutely or chronically in doses ranging from approximately 5 to 16.8 mmol (300–1041 mg), typically 2–3 h prior to exercise, and are recommended for athletes seeking to improve performance or endurance, particularly for exercise durations of around 10–17 min [73]. This range of nitrate intake aligns with current research and expert recommendations for both acute and chronic effects, demonstrating its safety and effectiveness [73]. However, it is noteworthy that for some participants, this dose exceeds the acceptable daily intake (ADI) of 3.7 mg/kg body weight set for the general (non-athletic) population [74]. Previous acute studies have reported increased urinary and fecal excretion of NOCs following similar high doses [75,76]. Additionally, unlike whole vegetables such as spinach or beetroot—which contain antioxidants like vitamin C that can inhibit NOC formation—commercial BJ supplements typically lack such protective compounds [72,77]. While our study focused on short-term outcomes, regular or chronic BJ supplementation, especially without concurrent antioxidant intake, may raise health concerns. Epidemiological data suggests a potential link between prolonged nitrate exposure and an increased risk of colorectal and gastric cancers [78,79].

Therefore, although BJ supplementation appears safe and beneficial in the context of short-term use among healthy, physically active individuals, its long-term safety should be evaluated carefully, particularly in non-athletic populations. Future research should consider population-specific factors such as health status, physical activity levels, dietary habits, and baseline antioxidant intake when determining optimal dosing strategies to maximize ergogenic effects while minimizing potential health risks.

### Strengths and Limitations

This study presents several strengths worth highlighting. It employed a double-blind, randomized crossover design, which enhances internal validity and reduces inter-individual variability, allowing for robust within-subject comparisons. The use of multiple, performance-relevant endpoints—including movement velocity, peak power, HRV, and SmO_2_—offers a comprehensive assessment of both the acute and short-term effects of BJ supplementation. Additionally, the inclusion of practical recovery metrics such as jump performance and DOMS provides ecologically valid outcomes that are directly applicable to real-world resistance training contexts.

Several limitations should also be acknowledged. The study’s small sample size (n = 11), which excluded females and non-physically active individuals, may limit statistical power and generalizability. Although our study exclusively included male participants, this decision was based on the goal of controlling for hormonal fluctuations that could potentially influence the outcomes, particularly in relation to nitrate metabolism and performance. Future research should aim to include both male and female participants to provide a more comprehensive understanding of the effects of BJ supplementation across genders. While efforts were made to ensure blinding, inherent sensory differences between BJ and the PLA might have compromised this process, introducing potential bias. Reliance on self-reported dietary and lifestyle compliance without biochemical verification (e.g., plasma nitrate/nitrite levels) further adds variability, without biochemical verification (e.g., plasma nitrate/nitrite levels or urinary NOCs), prevents confirmation of strict protocol adherence, and obscures individual variability in nitrate metabolism. Crucially, the absence of molecular biomarkers (e.g., oxidative stress markers like malondialdehyde, inflammatory cytokines, or DNA adducts such as O6-alkylguanine) limits mechanistic insights into BJ’s ergogenic effects and its potential to stimulate carcinogenic pathways. While studies demonstrated increased urinary NOC excretion following acute BJ intake [76], our design did not assess these outcomes, precluding a direct evaluation of safety risks alongside performance benefits.

Methodologically, the use of a Smith machine for the BS and BP, though enhancing safety and consistency, reduces ecological validity compared to that when free-weight training in real-world athletic settings is used. Similarly, semi-quantitative near-infrared spectroscopy (NIRS) and smartphone-based jump assessments, while pragmatic, lack the precision of laboratory-grade equipment (e.g., force plates and gas analyzers) and may underestimate subtle physiological changes. Moreover, while a 7-day washout period was implemented in accordance with Zhang et al. [68], residual beetroot effects cannot be entirely ruled out in the crossover design, and the focus on acute and 72 h recovery precludes insights into long-term supplementation benefits.

## 5. Conclusions

The current findings underscore BJ’s multifaceted ergogenic potential, across strength performance, cardiovascular regulation, autonomic function, and recovery. BJ intake appears to enhance both acute and short-term RT outcomes, particularly in high-intensity, phosphagen-dependent tasks. Its benefits likely stem from the nitrate-mediated pathways, promoting improved muscle perfusion, oxygen utilization, and neuromuscular efficiency. Notably, the observed reductions in cardiovascular strain and improvements in HRV suggest favorable autonomic adaptations that may support both exercise tolerance and recovery. Additionally, BJ supplementation appears to accelerate neuromuscular recovery and attenuate muscle soreness, possibly through nitric oxide (NO)-related anti-inflammatory, antioxidative, and mitochondrial-enhancing effects.

Collectively, these outcomes position BJ as a practical, time-sensitive nutritional strategy for athletes seeking to optimize training performance and recovery during periods of intense resistance exercise. For practitioners, we recommend (i) timing BJ intake 2–3 h pre-exercise to align with peak plasma nitrate availability, (ii) pairing acute BJ supplementation (e.g., 450 mg nitrate/day) with antioxidant-rich foods (e.g., citrus fruits) to mitigate potential NOC-related risks, and (iii) reserving chronic use for targeted training phases until long-term safety data are established.

Future studies incorporating direct assessments of plasma nitrate/nitrite concentrations and mechanistic biomarkers are warranted to validate these physiological effects and further refine BJ supplementation protocols for athletic populations. Critical research gaps remain, including investigations into BJ’s efficacy and safety in underrepresented cohorts (e.g., female athletes, older adults, and elite competitors), dose–response relationships across training modalities, and longitudinal monitoring of molecular biomarkers (e.g., oxidative stress, inflammatory cytokines, and DNA adducts) to evaluate carcinogenic risks. Additionally, comparative studies examining BJ’s effects in free-weight versus machine-based training environments could enhance ecological validity, while trials combining BJ with dietary nitrosation inhibitors (e.g., vitamin C, polyphenols) may optimize its risk–benefit profile.

## Figures and Tables

**Table 1:** GymBeam BIO beetroot powder nutrition facts.

Nutritional Value	100 g	15 g (Daily Dose)
**Values provided by GymBeam**		
**Energy value**	1305 kJ/310 kcal	196 kJ/46.5 kcal
**Fats**	0.7 g	0.11 g
**Saturated fats**	0 g	0 g
**Carbohydrates**	75 g	11 g
**Sugar**	53 g	8 g
**Protein**	12 g	1.8 g
**Salt**	0.36 g	0.05 g
**Vitamin B1**	5.6 g	0.84 g
**Iron**	37 g	0.55 g
**Mangan**	2.7 g	0.4 g
**Values provided by the Centre of Biotechnology of Sfax**		
**Sodium**	1510 mg	226.5 mg
**Potassium**	2690 mg	403.5 mg
**Magnesium**	210 mg	31.5 mg
**Chloride**	2260 mg	339 mg
**Nitrate**	3000 mg	450 mg
**Phosphate**	1060 mg	159 mg
**Sulfate**	360 mg	54 mg

**Table 2:** Dietary intake 24 h before sessions under beetroot juice (BJ) and placebo (PLA) conditions (mean ± SD).

Variable	BJ	PLA
**Energy (kcal)**	2450 ± 310	2380 ± 295
**Carbohydrates (g)**	312.4 ± 42.1	305.7 ± 39.8
**Protein (g)**	112.8 ± 18.6	110.5 ± 17.3
**Fat (g)**	87.1 ± 12.9	84.9 ± 11.7

**Table 3:** ∆Pre-Post change and ANOVA results in heart rate variability (HRV) indices and lactate recorded in two sessions for beetroot juice (BJ) and placebo (PLA) conditions.

	PLA		BJ		ANOVA Results
	1st Session	2nd Session	1st Session	2nd Session	
**MeanRR**	−26.48 ± 7.85	−15.6 ± 11.18 ^¤^	−25.6 ± 6.18	−14.43 ± 9.74 ^¤^	C: F(1, 10) = 0.13, *p* = 0.722, η^2^p = 0.013 **S: F(1, 10) = 25.39, *p* < 0.001, η^2^p = 0.717** C × S: F(1, 10) = 0.00, *p* = 0.961, η^2^p < 0.001
**RMSSD**	−40.78 ± 13.21	−19.01 ± 20.2 ^¤^	−32.74 ± 11.59 *	−19.66 ± 9.9 ^¤^	C: F(1, 10) = 0.86, *p* = 0.377, η^2^p = 0.079 **S: F(1, 10) = 17.10, *p* = 0.002, η^2^p = 0.631** C × S: F(1, 10) = 2.14, *p* = 0.174, η^2^p = 0.177
**SDNN**	−22.87 ± 16.74	−11.8 ± 16.94	−25.37 ± 8.24	−23.67 ± 12.92	C: F(1, 10) = 2.01, *p* = 0.187, η^2^p = 0.167 S: F(1, 10) = 3.46, *p* = 0.093, η^2^p = 0.257 C × S: F(1, 10) = 2.05, *p* = 0.183, η^2^p = 0.170
**LF**	−27.8 ± 19.6	−11.5 ± 5.72	−30.86 ± 15.11	−22.19 ± 16.92	C: F(1, 10) = 2.93, *p* = 0.118, η^2^p = 0.227 **S: F(1, 10) = 6.94, *p* = 0.025, η^2^p = 0.410** C × S: F(1, 10) = 0.66, *p* = 0.435, η^2^p = 0.062
**HF**	−34.56 ± 10.49	−15.09 ± 16.06 ^¤^	−23.08 ± 13.74	−11.98 ± 10.78 ^¤^	C: F(1, 10) = 1.49, *p* = 0.250, η^2^p = 0.130 **S: F(1, 10) = 21.54, *p* < 0.001, η^2^p = 0.683** C × S: F(1, 10) = 3.16, *p* = 0.106, η^2^p = 0.240
**Lactate**	135.24 ± 116.13	150.96 ± 78.74	182.02 ± 142.74	280.66 ± 247.96	C: F(1, 10) = 3.40, *p* = 0.095, η^2^p = 0.254 S: F(1, 10) = 1.17, *p* = 0.305, η^2^p = 0.105 C × S: F(1, 10) = 0.78, *p* = 0.397, η^2^p = 0.073

ANOVA: analysis of variance; C: condition; S: session; MeanRR: mean R–R interval; RMSSD: root mean square of successive differences; SDNN: standard Deviation of NN intervals; LF: low-frequency power; HF: high-frequency power; *: significantly different compared to PLA; ¤: significantly different compared to the 1st session. A *p* value of less than 0.05 was considered statistically significant for all analyses; Significant effects were highlighted in bold in the ANOVA results.

## Data Availability

The data supporting this study is available upon request from the corresponding author. The data are not publicly available due to privacy restrictions.

## Supplementary Materials

The following supporting information can be downloaded at https://www.mdpi.com/article/10.3390/nu17101720/s1: Table S1. Three-way ANOVA results for reached repetitions, peak velocity, peak power, peak HR, SmO_2_, and tHb during bench press and back squat.

## Abbreviations

The following abbreviations are used in this manuscript:

BJ	Beetroot Juice
PLA	Placebo
1RM	One-Repetition Maximum
BS	Back Squat
BP	Bench Press
HR	Heart Rate
HRV	Heart Rate Variability
RMSSD	Root Mean Square of Successive Differences
HF	High Frequency
CMJ	Countermovement Jump
SJ	Squat Jump
DOMS	Delayed Onset Muscle Soreness
RPE	Rate of Perceived Exertion
NO_3_^−^	Nitrate
NO_2_^−^	Nitrite
NO	Nitric Oxide
ATP	Adenosine Triphosphate
RT	Resistance Training
LF	Low Frequency
MeanRR	Mean RR Interval
SDNN	Standard Deviation of NN Intervals
SmO_2_	Muscle Oxygen Saturation
THb	Total Hemoglobin
NIRS	Near-Infrared Spectroscopy
∆Pre–Post	Delta Change from Pre- to Post-Intervention
η^2^p	Partial Eta-Squared
d	Cohen’s d (Effect Size)

## References

[1] Stoica F., Râpeanu G., Rațu R.N., Stănciuc N., Croitoru C., Țopa D., Jităreanu G. (2025). Red Beetroot and Its By-Products: A Comprehensive Review of Phytochemicals, Extraction Methods, Health Benefits, and Applications. Agriculture.

[2] Domínguez R., Cuenca E., Maté-Muñoz J.L., García-Fernández P., Serra-Paya N., Estevan M.C.L., Herreros P.V., Garnacho-Castaño M.V. (2017). Effects of beetroot juice supplementation on cardiorespiratory endurance in athletes. A systematic review. Nutrients.

[3] Lundberg J.O., Weitzberg E., Gladwin M.T. (2008). The nitrate–nitrite–nitric oxide pathway in physiology and therapeutics. Nat. Rev. Drug Discov..

[4] Bailey S.J., Fulford J., Vanhatalo A., Winyard P.G., Blackwell J.R., DiMenna F.J., Wilkerson D.P., Benjamin N., Jones A.M. (2010). Dietary nitrate supplementation enhances muscle contractile efficiency during knee-extensor exercise in humans. J. Appl. Physiol..

[5] Ferguson S.K., Hirai D.M., Copp S.W., Holdsworth C.T., Allen J.D., Jones A.M., Musch T.I., Poole D.C. (2013). Impact of dietary nitrate supplementation via beetroot juice on exercising muscle vascular control in rats. J. Physiol..

[6] Tan R., Pennell A., Karl S.T., Cass J.K., Go K., Clifford T., Bailey S.J., Perkins Storm C. (2023). Effects of dietary nitrate supplementation on back squat and bench press performance: A systematic review and meta-analysis. Nutrients.

[7] Mosher S.L., Sparks S.A., Williams E.L., Bentley D.J., Mc Naughton L.R. (2016). Ingestion of a nitric oxide enhancing supplement improves resistance exercise performance. J. Strength Cond. Res..

[8] Flanagan S.D., Looney D.P., Miller M.J., DuPont W.H., Pryor L., Creighton B.C., Sterczala A.J., Szivak T.K., Hooper D.R., Maresh C.M. (2016). The effects of nitrate-rich supplementation on neuromuscular efficiency during heavy resistance exercise. J. Am. Coll. Nutr..

[9] Siervo M., Lara J., Ogbonmwan I., Mathers J.C. (2013). Inorganic nitrate and beetroot juice supplementation reduces blood pressure in adults: A systematic review and meta-analysis. J. Nutr..

[10] Lansley K.E., Winyard P.G., Bailey S.J., Vanhatalo A., Wilkerson D.P., Blackwell J.R., Gilchrist M., Benjamin N., Jones A.M. (2011). Acute dietary nitrate supplementation improves cycling time trial performance. Med. Sci. Sports Exerc..

[11] Ranchal-Sanchez A., Diaz-Bernier V.M., De La Florida-Villagran C.A., Llorente-Cantarero F.J., Campos-Perez J., Jurado-Castro J.M. (2020). Acute effects of beetroot juice supplements on resistance training: A randomized double-blind crossover. Nutrients.

[12] Rodríguez-Fernández A., Castillo D., Raya-González J., Domínguez R., Bailey S.J. (2021). Beetroot juice supplementation increases concentric and eccentric muscle power output. J. Sci. Med. Sport.

[13] Rael B., Alfaro-Magallanes V.M., Romero-Parra N., Castro E.A., Cupeiro R., Janse de Jonge X.A., Wehrwein E.A., Peinado A.B., Group I.S. (2021). Menstrual cycle phases influence on cardiorespiratory response to exercise in endurance-trained females. Int. J. Environ. Res. Public Health.

[14] Williams T.D., Martin M.P., Mintz J.A., Rogers R.R., Ballmann C.G. (2020). Effect of acute beetroot juice supplementation on bench press power, velocity, and repetition volume. J. Strength Cond. Res..

[15] Jurado-Castro J.M., Casanova-Rodriguez D., Campos-Perez J., Llorente-Cantarero F.J., De La Florida-Villagran C.A., Diaz-Bernier V.M., Ranchal-Sanchez A. (2022). Beetroot Juice Produces Changes in Heart Rate Variability and Reduces Internal Load during Resistance Training in Men: A Randomized Double-Blind Crossover. Nutrients.

[16] Dong J.G. (2016). The role of heart rate variability in sports physiology. Exp. Ther. Med..

[17] Tekin R.T., Kudas S., Buran M.M., Cabuk S., Akbasli O., Uludag V., Yosmaoglu H.B. (2025). The relationship between resting heart rate variability and sportive performance, sleep and body awareness in soccer players. BMC Sports Sci. Med. Rehabil..

[18] Olas B. (2024). The cardioprotective role of nitrate-rich vegetables. Foods.

[19] Martinez M.W., Kim J.H., Shah A.B., Phelan D., Emery M.S., Wasfy M.M., Fernandez A.B., Bunch T.J., Dean P., Danielian A. (2021). Exercise-induced cardiovascular adaptations and approach to exercise and cardiovascular disease: JACC state-of-the-art review. J. Am. Coll. Cardiol..

[20] Brown L.E., Weir J.P. (2001). ASEP procedures recommendation I: Accurate assessment of muscular strength and power. J. Exerc. Physiol. Online.

[21] Conceição F., Fernandes J., Lewis M., Gonzaléz-Badillo J.J., Jimenéz-Reyes P. (2016). Movement velocity as a measure of exercise intensity in three lower limb exercises. J. Sports Sci..

[22] González-Badillo J.J., Sánchez-Medina L. (2010). Movement velocity as a measure of loading intensity in resistance training. Int. J. Sports Med..

[23] Morán-Navarro R., Pérez C.E., Mora-Rodríguez R., de la Cruz-Sánchez E., González-Badillo J.J., Sánchez-Medina L., Pallarés J.G. (2017). Time course of recovery following resistance training leading or not to failure. Eur. J. Appl. Physiol..

[24] Ammar A., Chtourou H., Trabelsi K., Padulo J., Turki M., El Abed K., Hoekelmann A., Hakim A. (2015). Temporal specificity of training: Intra-day effects on biochemical responses and Olympic-Weightlifting performances. J. Sports Sci..

[25] Ammar A., Turki M., Chtourou H., Hammouda O., Trabelsi K., Kallel C., Abdelkarim O., Hoekelmann A., Bouaziz M., Ayadi F. (2016). Pomegranate supplementation accelerates recovery of muscle damage and soreness and inflammatory markers after a weightlifting training session. PLoS ONE.

[26] Ammar A., Bailey S.J., Chtourou H., Trabelsi K., Turki M., Hökelmann A., Souissi N. (2018). Effects of pomegranate supplementation on exercise performance and post-exercise recovery in healthy adults: A systematic review. Br. J. Nutr..

[27] Ammar A., Chtourou H., Souissi N. (2017). Effect of time-of-day on biochemical markers in response to physical exercise. J. Strength Cond. Res..

[28] Gallardo E.J., Coggan A.R. (2019). What is in your beet juice? Nitrate and nitrite content of beet juice products marketed to athletes. Int. J. Sport Nutr. Exerc. Metab..

[29] Govoni M., Jansson E.Å., Weitzberg E., Lundberg J.O. (2008). The increase in plasma nitrite after a dietary nitrate load is markedly attenuated by an antibacterial mouthwash. Nitric Oxide.

[30] Sale C., Harris R.C., Florance J., Kumps A., Sanvura R., Poortmans J.R. (2009). Urinary creatine and methylamine excretion following 4 × 5g·day^−1^ or 20 × 1g·day^−1^ of creatine monohydrate for 5 days. J. Sports Sci..

[31] Sumaryanti S., Nugroho S., Visalim A., Ndayisenga J. (2022). Development of physical fitness test for mild intellectual disabilities aged 13-15 years. J. Keolahragaan.

[32] Feuerbacher J.F., Jacobs M.W., Dragutinovic B., Goldmann J.-P., Cheng S., Schumann M. (2023). Validity and test-retest reliability of the Vmaxpro sensor for evaluation of movement velocity in the deep squat. J. Strength Cond. Res..

[33] Vondrasek J.D., Riemann B.L., Grosicki G.J., Flatt A.A. (2024). Validity and efficacy of the elite HRV smartphone application during slow-paced breathing. Sensors.

[34] Boushel R., Piantadosi C. (2000). Near-infrared spectroscopy for monitoring muscle oxygenation. Acta Physiol. Scand..

[35] Crum E., O’Connor W., Van Loo L., Valckx M., Stannard S. (2017). Validity and reliability of the Moxy oxygen monitor during incremental cycling exercise. Eur. J. Sport Sci..

[36] Coswig V., Silva A.D.A.C.E., Barbalho M., De Faria F.R., Nogueira C.D., Borges M., Buratti J.R., Vieira I.B., Román F.J.L., Gorla J.I. (2019). Assessing the validity of the MyJUMP2 app for measuring different jumps in professional cerebral palsy football players: An experimental study. JMIR mHealth uHealth.

[37] Forsyth J., Farrally M. (2000). A comparison of lactate concentration in plasma collected from the toe, ear, and fingertip after a simulated rowing exercise. Br. J. Sports Med..

[38] Goodwin M.L., Harris J.E., Hernández A., Gladden L.B. (2007). Blood lactate measurements and analysis during exercise: A guide for clinicians. J. Diabetes Sci. Technol..

[39] Newham D., Mills K., Quigley B., Edwards R. (1983). Pain and fatigue after concentric and eccentric muscle contractions. Clin. Sci..

[40] R Core Team R. (2020). R: A Language and Environment for Statistical Computing.

[41] Cohen J. (2013). Statistical Power Analysis for the Behavioral Sciences.

[42] Hopkins W.G. (2002). A scale of magnitudes for effect statistics. New View Stat..

[43] Kassambara A. (2019). rstatix: Pipe-Friendly Framework for Basic Statistical Tests.

[44] Singmann H., Bolker B., Westfall J., Aust F. (2016). afex: Analysis of Factorial Experiments, version 0.16-1.

[45] Lenth R.V. (2016). Least-squares means: The R package lsmeans. J. Stat. Softw..

[46] Wickham H. (2016). ggplot2: Elegant Graphics for Data Analysis.

[47] Forbes S.P., Spriet L.L. (2022). Potential effect of beetroot juice supplementation on exercise economy in well-trained females. Appl. Physiol. Nutr. Metab..

[48] Głowacz J., Popiel M., Dziekoński K., Rybowski J., Krzyśkowska S., Redner A., Kwiecień I., Zwierzchlewska P., Rusin B., Stanibuła D. (2025). Beetroot Juice as a Natural Ergogenic Supplement: A Literature Review of Its Effects on Physical Performance and Training Adaptation. Qual. Sport.

[49] Dumar A.M., Huntington A.F., Rogers R.R., Kopec T.J., Williams T.D., Ballmann C.G. (2021). Acute beetroot juice supplementation attenuates morning-associated decrements in supramaximal exercise performance in trained sprinters. Int. J. Environ. Res. Public Health.

[50] Mueller B.J., Roberts M.D., Mobley C.B., Judd R.L., Kavazis A.N. (2024). Nitric oxide in exercise physiology: Past and present perspectives. Front. Physiol..

[51] Fulford J., Winyard P.G., Vanhatalo A., Bailey S.J., Blackwell J.R., Jones A.M. (2013). Influence of dietary nitrate supplementation on human skeletal muscle metabolism and force production during maximum voluntary contractions. Pflugers Arch..

[52] Phillips D.B., Brotto A.R., Ross B.A., Bryan T.L., Wong E.Y., Meah V.L., Fuhr D.P., van Diepen S., Stickland M.K., Network C.R.R. (2021). Inhaled nitric oxide improves ventilatory efficiency and exercise capacity in patients with mild COPD: A randomized-control cross-over trial. J. Physiol..

[53] Garnacho-Castaño M.V., Sánchez-Nuño S., Molina-Raya L., Carbonell T., Maté-Muñoz J.L., Pleguezuelos-Cobo E., Serra-Payá N. (2022). Circulating nitrate-nitrite reduces oxygen uptake for improving resistance exercise performance after rest time in well-trained CrossFit athletes. Sci. Rep..

[54] Crandall C.G., Wilson T.E. (2015). Human cardiovascular responses to passive heat stress. Compr. Physiol..

[55] Yuschen X., Choi J.-H., Seo J., Sun Y., Lee E., Kim S.-W., Park H.-Y. (2020). Effects of acute beetroot juice supplementation and exercise on cardiovascular function in healthy men in preliminary study: A randomized, double-blinded, placebo-controlled, and crossover trial. Healthcare.

[56] Larsen F.J., Weitzberg E., Lundberg J.O., Ekblom B. (2010). Dietary nitrate reduces maximal oxygen consumption while maintaining work performance in maximal exercise. Free Radic. Biol. Med..

[57] Jones A.M., Thompson C., Wylie L.J., Vanhatalo A. (2018). Dietary nitrate and physical performance. Annu. Rev. Nutr..

[58] Chen L., Zhu Y., Hu Z., Wu S., Jin C. (2021). Beetroot as a functional food with huge health benefits: Antioxidant, antitumor, physical function, and chronic metabolomics activity. Food Sci. Nutr..

[59] Jones L., Bailey S.J., Rowland S.N., Alsharif N., Shannon O.M., Clifford T. (2022). The effect of nitrate-rich beetroot juice on markers of exercise-induced muscle damage: A systematic review and meta-analysis of human intervention trials. J. Diet. Suppl..

[60] Ramanlal R., Gupta V. (2025). Physiology, Vasodilation. StatPearls.

[61] Benjamim C.J.R., de Sousa Júnior F.W., Porto A.A., Andrade C.V.G., de Figueiredo M.Í.L.S., Benjamim C.J.R., da Silva Rodrigues G., Rocha E.M.B., Cavalcante T.F., Garner D.M. (2023). Negligible Effects of Nutraceuticals from Beetroot Extract on Cardiovascular and Autonomic Recovery Response following Submaximal Aerobic Exercise in Physically Active Healthy Males: A Randomized Trial. Int. J. Environ. Res. Public Health.

[62] Gobbo H.R., Barbosa G.M., de Oliveira L.C., de Oliveira G.V. (2024). The Effect of Different Resistance Training Protocols on Cardiac Autonomic Modulation During Exercise Recovery: A Crossover, Randomized, and Controlled Pilot Study. J. Vasc. Dis..

[63] Daab W., Bouzid M.A., Lajri M., Bouchiba M., Saafi M.A., Rebai H. (2021). Chronic Beetroot Juice Supplementation Accelerates Recovery Kinetics following Simulated Match Play in Soccer Players. J. Am. Coll. Nutr..

[64] Hemmatinafar M., Zaremoayedi L., Koushkie Jahromi M., Alvarez-Alvarado S., Wong A., Niknam A., Suzuki K., Imanian B., Bagheri R. (2023). Effect of beetroot juice supplementation on muscle soreness and performance recovery after exercise-induced muscle damage in female volleyball players. Nutrients.

[65] Varshney K., Mishra K. (2022). An analysis of health benefits of beetroot. Int. J. Innov. Res. Eng. Manag..

[66] Gao C., Gupta S., Adli T., Hou W., Coolsaet R., Hayes A., Kim K., Pandey A., Gordon J., Chahil G. (2021). The effects of dietary nitrate supplementation on endurance exercise performance and cardiorespiratory measures in healthy adults: A systematic review and meta-analysis. J. Int. Soc. Sports Nutr..

[67] Rojano-Ortega D., Peña Amaro J., Berral-Aguilar A.J., Berral-de la Rosa F.J. (2022). Effects of Beetroot Supplementation on Recovery After Exercise-Induced Muscle Damage: A Systematic Review. Sports Health.

[68] Zhang J., Dai Z., Heung-Sang Wong S., Zheng C., Tsz-Chun Poon E. (2024). Acute effects of various doses of nitrate-rich beetroot juice on high-intensity interval exercise responses in women: A randomized, double-blinded, placebo-controlled, crossover trial. J. Int. Soc. Sports Nutr..

[69] Martinez R.M., Melo C.P.B., Pinto I.C., Mendes-Pierotti S., Vignoli J.A., Verri W.A., Casagrande R. (2024). Betalains: A Narrative Review on Pharmacological Mechanisms Supporting the Nutraceutical Potential Towards Health Benefits. Foods.

[70] Esen O., Domínguez R., Karayigit R. (2022). Acute Beetroot Juice Supplementation Enhances Intermittent Running Performance but Does Not Reduce Oxygen Cost of Exercise among Recreational Adults. Nutrients.

[71] Vermeer I.T.M., Van Maanen J. (2001). Nitrate exposure and endogenous formation of carcinogenic nitrosamines in humans. Rev. Environ. Health.

[72] IARC Working Group on the Evaluation of Carcinogenic Risks to Humans (2010). Ingested Nitrate and Nitrite, and Cyanobacterial Peptide Toxins. IARC Monographs on the Evaluation of Carcinogenic Risks to Humans.

[73] Macuh M., Knap B. (2021). Effects of Nitrate Supplementation on Exercise Performance in Humans: A Narrative Review. Nutrients.

[74] Karwowska M., Kononiuk A. (2020). Nitrates/Nitrites in Food-Risk for Nitrosative Stress and Benefits. Antioxidants.

[75] van Breda S.G., Mathijs K., Sági-Kiss V., Kuhnle G.G., Van der Veer B., Jones R.R., Sinha R., Ward M.H., de Kok T.M. (2019). Impact of high drinking water nitrate levels on the endogenous formation of apparent N-nitroso compounds in combination with meat intake in healthy volunteers. Environ. Health.

[76] Berends J.E., van den Berg L.M., Guggeis M.A., Henckens N.F., Hossein I.J., de Joode M.E., Zamani H., van Pelt K.A., Beelen N.A., Kuhnle G.G. (2019). Consumption of nitrate-rich beetroot juice with or without vitamin C supplementation increases the excretion of urinary nitrate, nitrite, and N-nitroso compounds in humans. Int. J. Mol. Sci..

[77] Bahadoran Z., Mirmiran P., Kabir A., Azizi F., Ghasemi A. (2017). The nitrate-independent blood pressure–lowering effect of beetroot juice: A systematic review and meta-analysis. Adv. Nutr..

[78] Ward M.H., Jones R.R., Brender J.D., De Kok T.M., Weyer P.J., Nolan B.T., Villanueva C.M., Van Breda S.G. (2018). Drinking water nitrate and human health: An updated review. Int. J. Environ. Res. Public Health.

[79] Zamani H., De Joode M., Hossein I., Henckens N., Guggeis M., Berends J., De Kok T., Van Breda S. (2021). The benefits and risks of beetroot juice consumption: A systematic review. Crit. Rev. Food Sci. Nutr..

