# Supplementary material for: Short-Term Beetroot Juice Supplementation Enhances Strength, Reduces Fatigue, and Promotes Recovery in Physically Active Individuals: A Randomized, Double-Blind, Crossover Trial
Source: Nutrients. 2025 May 19;17(10):1720. doi: 10.3390/nu17101720 (PMC12113839; doi:10.3390/nu17101720)
Supplement: Supplementary file 1 [file nutrients-17-01720-s001.zip › nutrients-3632501-supplementary.pdf]

Table S1. Three-way ANOVA results for reached repetitions, peak velocity, peak power, peak HR, SmO_2, and tHb during bench press and back squat.

		C	S	I	C × S	C × I	S × I	C × S × I
**Repetition**	**BP**	***$F(1, 10) = 17.06$<br>$p = 0.002$<br>$\eta^2p = 0.630$***	$F(1, 10) = 0.25$<br>$p = 0.630$<br>$\eta^2p = 0.024$	***$F(1.33, 13.27) = 209.05$<br>$p < 0.001$<br>$\eta^2p = 0.954$***	$F(1, 10) = 0.89$<br>$p = 0.368$<br>$\eta^2p = 0.082$	$F(1.39, 13.89) = 0.89$<br>$p = 0.395$<br>$\eta^2p = 0.082$	$F(1.58, 15.84) = 0.63$<br>$p = 0.510$<br>$\eta^2p = 0.059$	$F(1.17, 11.69) = 0.01$<br>$p = 0.945$<br>$\eta^2p = 0.001$
	**BS**	***$F(1, 10) = 10.95$<br>$p = 0.008$<br>$\eta^2p = 0.523$***	***$F(1, 10) = 5.12$<br>$p = 0.047$<br>$\eta^2p = 0.339$***	***$F(1.31, 13.14) = 56.20$<br>$p < 0.001$<br>$\eta^2p = 0.849$***	$F(1, 10) = 1.78$<br>$p = 0.212$<br>$\eta^2p = 0.151$	$F(1.89, 18.93) = 2.77$<br>$p = 0.091$<br>$\eta^2p = 0.217$	$F(1.44, 14.40) = 0.02$<br>$p = 0.956$<br>$\eta^2p = 0.002$	$F(1.95, 19.47) = 0.04$<br>$p = 0.963$<br>$\eta^2p = 0.003$
**Peak velocity**	**BP**	***$F(1, 10) = 220.78$<br>$p < 0.001$<br>$\eta^2p = 0.957$***	***$F(1, 10) = 11.53$<br>$p = 0.007$<br>$\eta^2p = 0.536$***	***$F(1.93, 19.33) = 103.15$<br>$p < 0.001$<br>$\eta^2p = 0.912$***	$F(1, 10) = 0.02$<br>$p = 0.891$<br>$\eta^2p = 0.002$	$F(1.76, 17.63) = 0.22$<br>$p = 0.778$<br>$\eta^2p = 0.021$	$F(1.85, 18.47) = 1.84$<br>$p = 0.188$<br>$\eta^2p = 0.156$	$F(1.42, 14.22) = 1.51$<br>$p = 0.249$<br>$\eta^2p = 0.131$
	**BS**	***$F(1, 10) = 87.65$<br>$p < 0.001$<br>$\eta^2p = 0.898$***	***$F(1, 10) = 13.48$<br>$p = 0.004$<br>$\eta^2p = 0.574$***	***$F(1.76, 17.65) = 106.80$<br>$p < 0.001$<br>$\eta^2p = 0.914$***	$F(1, 10) = 0.08$<br>$p = 0.786$<br>$\eta^2p = 0.008$	$F(1.75, 17.53) = 0.13$<br>$p = 0.856$<br>$\eta^2p = 0.013$	$F(1.44, 14.44) = 1.49$<br>$p = 0.254$<br>$\eta^2p = 0.129$	$F(1.94, 19.41) = 0.07$<br>$p = 0.931$<br>$\eta^2p = 0.007$
**Peak power**	**BP**	$F(1, 10) = 0.20$<br>$p = 0.663$<br>$\eta^2p = 0.020$	$F(1, 10) = 0.15$<br>$p = 0.703$<br>$\eta^2p = 0.015$	***$F(1.88, 18.80) = 277.44$<br>$p < 0.001$<br>$\eta^2p = 0.965$***	$F(1, 10) = 0.28$<br>$p = 0.609$<br>$\eta^2p = 0.027$	$F(1.60, 16.04) = 0.72$<br>$p = 0.473$<br>$\eta^2p = 0.067$	$F(1.27, 12.71) = 0.30$<br>$p = 0.650$<br>$\eta^2p = 0.029$	$F(1.92, 19.25) = 0.11$<br>$p = 0.894$<br>$\eta^2p = 0.010$
	**BS**	$F(1, 10) = 1.36$<br>$p = 0.271$<br>$\eta^2p = 0.119$	$F(1, 10) = 1.63$<br>$p = 0.231$<br>$\eta^2p = 0.140$	***$F(1.85, 18.51) = 565.67$<br>$p < 0.001$<br>$\eta^2p = 0.983$***	$F(1, 10) = 0.90$<br>$p = 0.366$<br>$\eta^2p = 0.082$	$F(1.52, 15.22) = 0.59$<br>$p = 0.523$<br>$\eta^2p = 0.056$	$F(1.79, 17.91) = 0.79$<br>$p = 0.455$<br>$\eta^2p = 0.074$	***$F(1.64, 16.41) = 4.60$<br>$p = 0.032$<br>$\eta^2p = 0.315$***
**Peak HR**	**BP**	***$F(1, 10) = 250.20$<br>$p < 0.001$<br>$\eta^2p = 0.962$***	$F(1, 10) = 0.24$<br>$p = 0.635$<br>$\eta^2p = 0.023$	***$F(1.88, 18.83) = 325.79$<br>$p < 0.001$<br>$\eta^2p = 0.970$***	$F(1, 10) = 0.08$<br>$p = 0.782$<br>$\eta^2p = 0.008$	***$F(1.70, 17.05) = 16.29$<br>$p < 0.001$<br>$\eta^2p = 0.620$***	$F(1.80, 18.00) = 2.83$<br>$p = 0.090$<br>$\eta^2p = 0.221$	$F(1.78, 17.78) = 0.14$<br>$p = 0.846$<br>$\eta^2p = 0.014$
	**BS**	***$F(1, 10) = 5.76$<br>$p = 0.037$<br>$\eta^2p = 0.366$***	$F(1, 10) = 0.43$<br>$p = 0.525$<br>$\eta^2p = 0.042$	***$F(1.89, 18.90) = 31.21$<br>$p < 0.001$<br>$\eta^2p = 0.757$***	$F(1, 10) = 1.08$<br>$p = 0.323$<br>$\eta^2p = 0.097$	$F(1.44, 14.42) = 0.67$<br>$p = 0.478$<br>$\eta^2p = 0.063$	$F(1.52, 15.24) = 1.17$<br>$p = 0.321$<br>$\eta^2p = 0.105$	***$F(1.70, 17.03) = 4.12$<br>$p = 0.040$<br>$\eta^2p = 0.292$***
**SmO_2**	**BP**	***$F(1, 10) = 128.93$<br>$p < 0.001$<br>$\eta^2p = 0.928$:***	***$F(1, 10) = 8.90$<br>$p = 0.014$<br>$\eta^2p = 0.471$***	$F(1.81, 18.05) = 2.10$<br>$p = 0.154$<br>$\eta^2p = 0.174$	***$F(1, 10) = 22.71$<br>$p < 0.001$<br>$\eta^2p = 0.694$***	$F(1.59, 15.85) = 0.31$<br>$p = 0.689$<br>$\eta^2p = 0.030$	$F(1.68, 16.80) = 0.41$<br>$p = 0.637$<br>$\eta^2p = 0.039$	$F(1.83, 18.33) = 3.21$<br>$p = 0.068$<br>$\eta^2p = 0.243$
	**BS**	***$F(1, 10) = 89.69$<br>$p < 0.001$<br>$\eta^2p = 0.900$***	***$F(1, 10) = 27.1$<br>$p < 0.001$<br>$\eta^2p = 0.731$***	$F(1.84, 18.43) = 1.91$<br>$p = 0.178$<br>$\eta^2p = 0.160$	***$F(1, 10) = 38.93$<br>$p < 0.001$<br>$\eta^2p = 0.796$***	$F(1.56, 15.57) = 0.18$<br>$p = 0.782$<br>$\eta^2p = 0.018$	$F(1.60, 15.98) = 0.52$<br>$p = 0.564$<br>$\eta^2p = 0.050$	$F(1.49, 14.86) = 0.11$<br>$p = 0.839$<br>$\eta^2p = 0.011$
**tHb**	**BP**	***$F(1, 10) = 6.46$<br>$p = 0.029$<br>$\eta^2p = 0.392$***	$F(1, 10) = 1.89$<br>$p = 0.199$<br>$\eta^2p = 0.159$	$F(1.48, 14.83) = 0.57$<br>$p = 0.529$<br>$\eta^2p = 0.054$	$F(1, 10) = 1.93$<br>$p = 0.195$<br>$\eta^2p = 0.161$	$F(1.75, 17.49) = 3.17$<br>$p = 0.072$<br>$\eta^2p = 0.241$	$F(1.80, 18.03) = 0.80$<br>$p = 0.453$<br>$\eta^2p = 0.074$	$F(1.83, 18.31) = 0.08$<br>$p = 0.906$<br>$\eta^2p = 0.008$
	**BS**	$F(1, 10) = 0.69$<br>$p = 0.427$<br>$\eta^2p = 0.064$	$F(1, 10) = 0.01$<br>$p = 0.922$<br>$\eta^2p = 0.001$	$F(1.76, 17.62) = 0.66$<br>$p = 0.513$<br>$\eta^2p = 0.062$	$F(1, 10) = 0.55$<br>$p = 0.475$<br>$\eta^2p = 0.052$	$F(1.60, 16.04) = 0.07$<br>$p = 0.898$<br>$\eta^2p = 0.007$	***$F(1.37, 13.70) = 4.93$<br>$p = 0.035$<br>$\eta^2p = 0.330$***	$F(1.83, 18.31) = 1.63$<br>$p = 0.224$<br>$\eta^2p = 0.140$

HR: Heart rate; SmO_2: Muscle oxygenation; tHb: Total haemoglobin; BP: Bench press; BS: Back Squat; C: Condition; S: Session; I: Intensity. Significant effects were highlighted in bold.
